# *catena*-Poly[[diaqua­cadmium(II)]-μ-(methyl morpholino dichloro­methylene­diphospho­nato)-κ^3^ *O*,*O*′:*O*′′-[tetra­aqua­cadmium(II)]-μ-(methyl morpholino dichloro­methyl­enediphospho­nato)-κ^3^ *O*:*O*′*,O*′′]

**DOI:** 10.1107/S160053680901527X

**Published:** 2009-04-30

**Authors:** Jonna Jokiniemi, Jouko Vepsäläinen, Markku Ahlgrén

**Affiliations:** a Department of Chemistry, University of Joensuu, PO Box 111, FI-80101 Joensuu, Finland; b Laboratory of Chemistry, Department of Biosciences, University of Kuopio, PO Box 1627, FI-70211 Kuopio, Finland

## Abstract

The asymmetric unit of the title compound, [Cd(C_6_H_11_Cl_2_NO_6_P_2_)(H_2_O)_3_]_*n*_, contains two octahedrally coordinated Cd atoms located in special positions, one on a twofold rotation axis and the other on a centre of symmetry. The metal atoms are connected by methyl morpholino dichloro­methyl­enediphospho­nate ligands into chains in the *c-*axis direction. These chains are further connected by O—H⋯O hydrogen bonds into a layer-like construction along (100).

## Related literature

For applications of metal complexes of bis­phospho­nates, see: Clearfield (1998 ▶); Clearfield *et al.* (2001 ▶); Fu *et al.* (2007 ▶). For cadmium bis­phospho­nate complexes, see: Ying & Mao (2006 ▶); Man *et al.* (2006 ▶). For metal complexes of bis­phospho­nate ester derivatives, see: Jokiniemi *et al.* (2007 ▶, 2008 ▶). For Mg, Zn and Cd complexes of the symmetrical diethyl ester derivative of (dichloro­methyl­ene)bis­phospho­nate, see: Kontturi *et al.* (2002 ▶, 2005*a* ▶,*b* ▶).
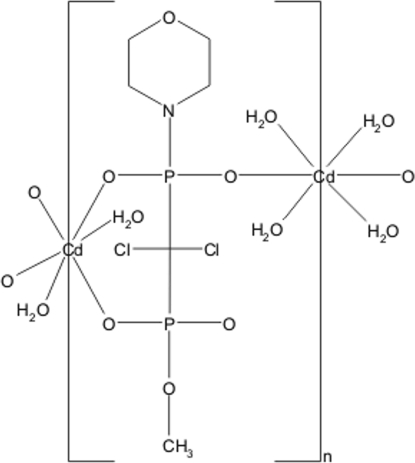


## Experimental

### 

#### Crystal data

[Cd(C_6_H_11_Cl_2_NO_6_P_2_)(H_2_O)_3_]
*M* *_r_* = 492.45
Monoclinic, 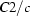

*a* = 26.2488 (8) Å
*b* = 7.6578 (3) Å
*c* = 17.5445 (7) Å
β = 116.002 (3)°
*V* = 3169.6 (2) Å^3^
*Z* = 8
Mo *K*α radiation
μ = 1.96 mm^−1^
*T* = 120 K
0.30 × 0.25 × 0.20 mm

#### Data collection

Nonius KappaCCD diffractometer
Absorption correction: multi-scan (*XPREP* in *SHELXTL*; Sheldrick, 2008 ▶) *T*_min_ = 0.565, *T*_max_ = 0.676
21828 measured reflections
4053 independent reflections
3370 reflections with *I* > 2σ(*I*)
*R*_int_ = 0.038

#### Refinement

*R*[*F* ^2^ > 2σ(*F* ^2^)] = 0.027
*wR*(*F* ^2^) = 0.068
*S* = 1.06
4053 reflections
195 parameters
H-atom parameters constrained
Δρ_max_ = 1.04 e Å^−3^
Δρ_min_ = −1.12 e Å^−3^

### 

Data collection: *COLLECT* (Nonius, 1997 ▶); cell refinement: *DENZO*/*SCALEPACK* (Otwinowski & Minor, 1997 ▶); data reduction: *DENZO*/*SCALEPACK*; program(s) used to solve structure: *SHELXS97* (Sheldrick, 2008 ▶); program(s) used to refine structure: *SHELXL97* (Sheldrick, 2008 ▶); molecular graphics: *DIAMOND* (Brandenburg, 2005 ▶); software used to prepare material for publication: *SHELXL97*.

## Supplementary Material

Crystal structure: contains datablocks I, global. DOI: 10.1107/S160053680901527X/er2063sup1.cif

Structure factors: contains datablocks I. DOI: 10.1107/S160053680901527X/er2063Isup2.hkl

Additional supplementary materials:  crystallographic information; 3D view; checkCIF report

## Figures and Tables

**Table 1:** Selected geometric parameters (Å, °)

Cd1—O11	2.2256 (17)
Cd1—O21	2.3173 (16)
Cd1—O1	2.3409 (17)
Cd2—O12	2.1884 (17)
Cd2—O3	2.2795 (16)
Cd2—O2	2.3486 (16)

**Table 2:** Hydrogen-bond geometry (Å, °)

*D*—H⋯*A*	*D*—H	H⋯*A*	*D*⋯*A*	*D*—H⋯*A*
O1—H1*A*⋯O2^i^	0.84	2.06	2.849 (2)	156
O1—H1*B*⋯O22^ii^	0.88	2.12	2.990 (2)	170
O2—H2*A*⋯O21^iii^	0.86	2.04	2.844 (2)	155
O2—H2*B*⋯O22	0.86	1.84	2.662 (2)	159
O3—H3*A*⋯O22	0.83	2.03	2.773 (2)	149
O3—H3*B*⋯O13^iv^	0.90	1.87	2.745 (2)	163

Symmetry codes: (i) 
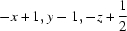
; (ii) 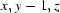; (iii) 
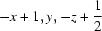
; (iv) 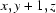.

**Fig. 1.**
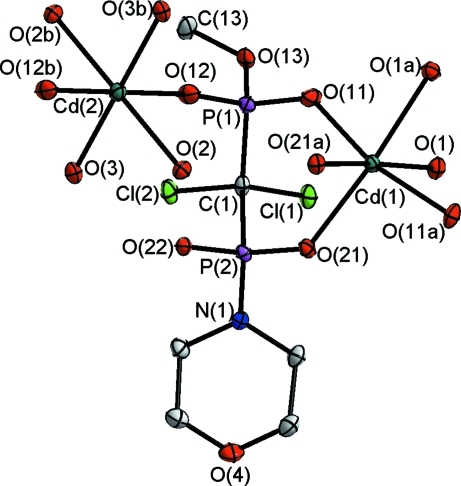
Structure of the title compound showing the atomic numbering scheme and 50% probability displacement ellipsoids. Hydrogen atoms are omitted for clarity. Atoms labelled with suffixes A and B are at the symmetry postitions (1 - x, y, 1/2 - z) and (1 - x, 1 - y, - z) respectively.

**Fig. 2.**
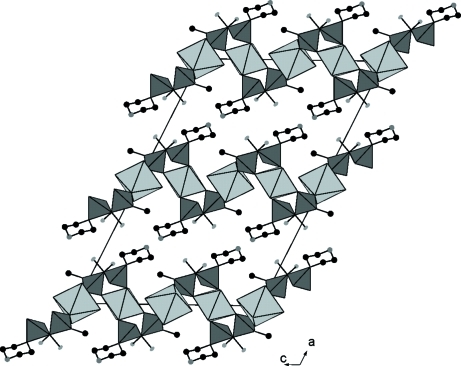
Packing of the title compound viewed along the b-axis. CdO6 octahedra are presented in medium grey and PO3C and NPO2C tetrahedra in dark grey. Hydrogen atoms are omitted for clarity.

## supplementary crystallographic information

### Comment

Metal complexes with bisphosphonic acids have interesting structures with various coordination architectures, and properties that offer practical applications in catalysis, ion-exchange and sorption (Clearfield *et al.*, 2001, Clearfield, 1998, Fu *et al.*, 2007). In our recent investigations, we studied the complexing properties of amide ester derivatives of (dichloromethylene)bisphosphonate, Cl_2_MBP (Jokiniemi *et al.*, 2007, 2008). Introduction of these ester substituents to phosphorus groups can result in novel structures of metal bishosphonates and lead to interesting functionalities. We now present the crystal structure of the Cd(II) complex of the *P*-morpholinyl-*P*'-methyl ester derivative of Cl_2_MBP obtained by gel crystallization.

The title compound is isomorphous with the earlier reported Mg complexes of (dichloromethylene)bisphosphonic acid methyl esters of piperidinyl and morpholinyl derivatives (Jokiniemi *et al.*, 2007, 2008). The title compound is polymeric, consisting of chains in the direction of the *c*-axis. There are two crystallographically independent six-coordinated Cd^2+^ cations in the asymmetric unit, located in special positions: Cd1 on the twofold rotation axis and Cd2 on the centre of symmetry (Fig. 1). Two symmetrically related *L*_1_ ligands, *L*_1_ = (Cl_2_CP2O_5_MeNC_4_H_8_O), around the Cd1 atom form six-membered chelate rings. The *L*_1_ ligand is further connected to Cd2 through one O atom, and thus acts as a triatomic bridge between the adjacent Cd atoms. The fourth phosphonate O atom remains non-coordinated but is involved in hydrogen bonding. The remaining coordination sites around the Cd1 atoms are occupied by aqua ligands in *cis* position; the geometry is a significantly distorted octahedron having Cd1–O bond distances 2.226 (2)–2.341 (2) Å (Table 1). The three *trans* angles are O11–Cd1–O11A 166.14 (9), O21–Cd1–O1A 174.23 (5) and O21A–Cd1–O1 174.23 (5)°. The Cd2 atom has a distorted octahedral geometry, and the binding sites around the metal atom are occupied by two phosphonate O atoms in axial positions and four aqua ligands having Cd2–O bond lengths 2.188 (2)–2.349 (2) Å. The three *trans* bond angles are 180°, while the *cis* bond angles around the Cd2 atom range from 82.50 (6) to 97.50 (6)°. In addition to isomorphous Mg complexes of monomethyl ester of morpholinyl and piperidinyl derivatives of Cl_2_MBP (Jokiniemi *et al.*, 2007 and 2008), the same kind of chain construction is found in Mg, Zn and Cd complexes of the symmetrical diethyl ester derivative of Cl_2_MBP (Kontturi *et al.*, 2002, 2005a and 2005b).

The polymeric chains are connected, in a layer-like structure parallel to the (100) plane, by hydrogen bonds [O···O 2.745 (2)–2.990 (2) Å, 149–170°, Table 2]. The morpholinyl rings and chlorine atoms of the *L*_1_ ligands point out from the layers (Fig. 2), which are held together solely by weak Van der Waals interactions, with an interlayer distance of 11.7959 Å.

### Experimental

(H_2_N[(CH_2_)_2_]_2_O)_2_CH_3_PO_3_CCl_2_PO_2_NC_4_H_8_O (19.8 mg, 0.039 mmol) and Cd(NO_3_)_2_×4H_2_O (12.1 mg, 0.039 mmol) were dissolved separately in water (0.45 ml), the solutions were mixed, and tetramethoxysilane (TMOS 0.1 ml) was added. The two-phase system was shaken until homogeneous. After gel formation, a precipitant, acetone (1.0 ml), was added above the gel to induce crystallization. After about three weeks, large, colourless plank-shaped crystals suitable for X-ray analysis formed at the gel-liquid boundary. Anal. Found: C, 14.63; H, 3.48; N, 2.84; Cd, 22.83%. Calc. for C_6_H_17_Cl_2_CdNO_9_P_2_: C, 14.74; H, 3.48; N, 2.86; Cd, 22.45%. Main IR absorptions (KBr pellet, cm^-1^): 3432 (*s*), 2961 (*m*), 2926 (*m*), 2854 (*m*), 1627 (*m*), 1204 (*versus*), 1145 (*m*), 1101 (*versus*), 1072 (*s*), 1056 (*s*), 981 (*s*), 869 (*m*), 843 (*m*). ^31^P CP/MAS NMR: δ_P_ 8.4 and 4.3 p.p.m.. TGA (25–900 °C under a synthetic air): 30–110 °C 12.6% (calculated 11.0% for the loss of three aqua ligands). The second step (190–900 °C) is attributed to the release of organic groups, chlorine atoms and a methylene carbon atom. The observed total weight loss is 47.0% (calculated 45.1% if the final product is assumed to be Cd(PO_3_)_2_).

### Refinement

H atoms of the methyl and morpholinyl groups were placed at calculated positions in the riding-model approximation with C–H = 0.99 Å (morpholinyl) [*U*_ISO_(H) = 1.2*U*_eq_(C)] and C–H = 0.98 Å (methyl) [*U*_ISO_(H) = 1.5*U*_eq_(C)]. H atoms of the aqua ligands were located in a difference map and treated as riding, with O–H bond lengths constrained to 0.83–0.90 Å and with *U*_ISO_(H) = 1.5*U*_eq_(O).

### Crystal data

**Table:**

[Cd(C_6_H_11_Cl_2_NO_6_P_2_)(H_2_O)_3_]	*F*(000) = 1952
*M**_r_* = 492.45	*D*_x_ = 2.064 Mg m^−^^3^
Monoclinic, *C*2/*c*	Mo *K*α radiation, λ = 0.71073 Å
Hall symbol: -C 2yc	Cell parameters from 21828 reflections
*a* = 26.2488 (8) Å	θ = 2.8–28.7°
*b* = 7.6578 (3) Å	µ = 1.96 mm^−^^1^
*c* = 17.5445 (7) Å	*T* = 120 K
β = 116.002 (3)°	Plank, colourles
*V* = 3169.6 (2) Å^3^	0.30 × 0.25 × 0.20 mm
*Z* = 8	

### Data collection

**Table:**

Nonius KappaCCD diffractometer	4053 independent reflections
Radiation source: fine-focus sealed tube	3370 reflections with *I* > 2σ(*I*)
graphite	*R*_int_ = 0.038
multi–scan	θ_max_ = 28.7°, θ_min_ = 2.8°
Absorption correction: multi-scan (*XPREP* in *SHELXTL*; Sheldrick, 2008)	*h* = −35→35
*T*_min_ = 0.565, *T*_max_ = 0.676	*k* = −10→10
21828 measured reflections	*l* = −23→21

### Refinement

**Table:**

Refinement on *F*^2^	Secondary atom site location: difference Fourier map
Least-squares matrix: full	Hydrogen site location: inferred from neighbouring sites
*R*[*F*^2^ > 2σ(*F*^2^)] = 0.027	H-atom parameters constrained
*wR*(*F*^2^) = 0.068	*w* = 1/[σ^2^(*F*_o_^2^) + (0.04*P*)^2^] where *P* = (*F*_o_^2^ + 2*F*_c_^2^)/3
*S* = 1.06	(Δ/σ)_max_ < 0.001
4053 reflections	Δρ_max_ = 1.03 e Å^−^^3^
195 parameters	Δρ_min_ = −1.12 e Å^−^^3^
0 restraints	Extinction correction: *SHELXL97* (Sheldrick, 2008), Fc^*^=kFc[1+0.001xFc^2^λ^3^/sin(2θ)]^-1/4^
Primary atom site location: structure-invariant direct methods	Extinction coefficient: 0.00053 (7)

### Special details

**Table:**

Geometry. All e.s.d.'s (except the e.s.d. in the dihedral angle between two l.s. planes) are estimated using the full covariance matrix. The cell e.s.d.'s are taken into account individually in the estimation of e.s.d.'s in distances, angles and torsion angles; correlations between e.s.d.'s in cell parameters are only used when they are defined by crystal symmetry. An approximate (isotropic) treatment of cell e.s.d.'s is used for estimating e.s.d.'s involving l.s. planes.
Refinement. Refinement of *F*^2^ against ALL reflections. The weighted *R*-factor *wR* and goodness of fit *S* are based on *F*^2^, conventional *R*-factors *R* are based on *F*, with *F* set to zero for negative *F*^2^. The threshold expression of *F*^2^ > σ(*F*^2^) is used only for calculating *R*-factors(gt) *etc*. and is not relevant to the choice of reflections for refinement. *R*-factors based on *F*^2^ are statistically about twice as large as those based on *F*, and *R*- factors based on ALL data will be even larger.

### Fractional atomic coordinates and isotropic or equivalent isotropic displacement parameters (Å^2^)

**Table:**

	*x*	*y*	*z*	*U*_iso_*/*U*_eq_	
Cl1	0.33755 (3)	0.03444 (8)	0.08713 (4)	0.01735 (14)	
Cl2	0.31951 (3)	0.31865 (8)	−0.03219 (3)	0.01910 (14)	
P1	0.42845 (3)	0.12553 (8)	0.03696 (4)	0.01308 (14)	
P2	0.39459 (2)	0.37248 (8)	0.15068 (4)	0.01129 (13)	
Cd1	0.5000	0.05298 (3)	0.2500	0.01210 (8)	
Cd2	0.5000	0.5000	0.0000	0.01366 (8)	
O1	0.43819 (7)	−0.1737 (2)	0.24266 (10)	0.0170 (4)	
H1A	0.4474	−0.2385	0.2856	0.025*	
H1B	0.4298	−0.2517	0.2023	0.025*	
O2	0.52362 (7)	0.5362 (2)	0.14457 (10)	0.0149 (4)	
H2A	0.5420	0.4483	0.1748	0.022*	
H2B	0.4929	0.5313	0.1507	0.022*	
O3	0.41722 (7)	0.6409 (2)	−0.02767 (10)	0.0182 (4)	
H3A	0.4041	0.6034	0.0045	0.027*	
H3B	0.4177	0.7579	−0.0243	0.042 (9)*	
O4	0.26897 (8)	0.6093 (3)	0.22336 (12)	0.0281 (4)	
O11	0.46611 (8)	0.0179 (2)	0.11038 (11)	0.0182 (4)	
O12	0.45203 (8)	0.2725 (2)	0.00769 (11)	0.0220 (4)	
O13	0.39645 (7)	−0.0065 (2)	−0.03937 (10)	0.0172 (4)	
O21	0.43465 (7)	0.2737 (2)	0.22805 (9)	0.0138 (3)	
O22	0.41655 (7)	0.5285 (2)	0.12215 (10)	0.0142 (4)	
N1	0.33955 (8)	0.4329 (3)	0.16396 (12)	0.0147 (4)	
C1	0.37131 (10)	0.2129 (3)	0.06118 (14)	0.0130 (5)	
C2	0.29966 (10)	0.5686 (3)	0.11230 (15)	0.0187 (5)	
H2E	0.2634	0.5141	0.0731	0.022*	
H2F	0.3155	0.6307	0.0781	0.022*	
C3	0.28936 (12)	0.6969 (4)	0.16995 (17)	0.0246 (6)	
H3E	0.3251	0.7585	0.2056	0.029*	
H3F	0.2612	0.7851	0.1351	0.029*	
C5	0.30950 (12)	0.4819 (4)	0.27479 (17)	0.0240 (6)	
H5E	0.2955	0.4245	0.3125	0.029*	
H5F	0.3457	0.5408	0.3109	0.029*	
C6	0.31968 (11)	0.3450 (3)	0.22048 (15)	0.0188 (5)	
H6E	0.3485	0.2601	0.2570	0.023*	
H6F	0.2841	0.2808	0.1865	0.023*	
C13	0.37843 (13)	0.0444 (4)	−0.12767 (15)	0.0267 (6)	
H13A	0.3832	0.1707	−0.1308	0.040*	
H13B	0.3384	0.0137	−0.1610	0.040*	
H13C	0.4015	−0.0171	−0.1504	0.040*	

### Atomic displacement parameters (Å^2^)

**Table:**

	*U*^11^	*U*^22^	*U*^33^	*U*^12^	*U*^13^	*U*^23^
Cl1	0.0178 (3)	0.0143 (3)	0.0189 (3)	−0.0048 (2)	0.0070 (2)	0.0009 (2)
Cl2	0.0207 (3)	0.0164 (3)	0.0133 (3)	0.0014 (2)	0.0010 (2)	0.0018 (2)
P1	0.0172 (3)	0.0098 (3)	0.0123 (3)	−0.0019 (2)	0.0065 (2)	−0.0020 (2)
P2	0.0116 (3)	0.0097 (3)	0.0117 (3)	−0.0005 (2)	0.0042 (2)	−0.0006 (2)
Cd1	0.01370 (13)	0.00967 (13)	0.01166 (13)	0.000	0.00440 (9)	0.000
Cd2	0.01748 (14)	0.01108 (14)	0.01369 (14)	−0.00167 (9)	0.00800 (10)	−0.00030 (9)
O1	0.0202 (9)	0.0148 (9)	0.0144 (8)	−0.0028 (7)	0.0062 (7)	0.0010 (7)
O2	0.0155 (9)	0.0161 (9)	0.0129 (9)	0.0000 (7)	0.0060 (7)	0.0004 (7)
O3	0.0229 (10)	0.0124 (9)	0.0210 (9)	0.0016 (7)	0.0112 (7)	0.0029 (7)
O4	0.0294 (11)	0.0286 (11)	0.0380 (11)	0.0081 (9)	0.0254 (9)	0.0052 (9)
O11	0.0201 (9)	0.0220 (10)	0.0104 (9)	0.0041 (7)	0.0047 (7)	−0.0021 (7)
O12	0.0316 (11)	0.0129 (9)	0.0309 (10)	−0.0063 (8)	0.0225 (9)	−0.0042 (7)
O13	0.0257 (10)	0.0107 (9)	0.0117 (9)	−0.0015 (7)	0.0050 (7)	−0.0020 (6)
O21	0.0137 (8)	0.0132 (9)	0.0123 (8)	0.0013 (6)	0.0036 (6)	−0.0002 (6)
O22	0.0165 (9)	0.0120 (9)	0.0165 (9)	−0.0007 (7)	0.0094 (7)	0.0003 (6)
N1	0.0172 (11)	0.0143 (11)	0.0150 (10)	0.0018 (8)	0.0092 (8)	0.0031 (8)
C1	0.0142 (11)	0.0108 (12)	0.0111 (11)	−0.0021 (9)	0.0030 (9)	0.0015 (9)
C2	0.0159 (12)	0.0208 (14)	0.0187 (13)	0.0049 (10)	0.0071 (10)	0.0043 (10)
C3	0.0276 (15)	0.0210 (14)	0.0301 (15)	0.0069 (11)	0.0173 (12)	0.0027 (11)
C5	0.0303 (16)	0.0247 (15)	0.0247 (15)	−0.0005 (11)	0.0192 (12)	0.0025 (11)
C6	0.0202 (13)	0.0185 (13)	0.0212 (13)	−0.0030 (10)	0.0124 (10)	0.0025 (10)
C13	0.0457 (18)	0.0198 (14)	0.0107 (13)	0.0026 (12)	0.0088 (12)	−0.0007 (10)

### Geometric parameters (Å, °)

**Table:**

Cl1—C1	1.792 (2)	O1—H1B	0.8775
Cl2—C1	1.799 (2)	O2—H2A	0.8617
P1—O12	1.4803 (18)	O2—H2B	0.8598
P1—O11	1.4829 (18)	O3—H3A	0.8298
P1—O13	1.5922 (17)	O3—H3B	0.8982
P1—C1	1.854 (2)	O4—C3	1.433 (3)
P2—O22	1.5044 (17)	O4—C5	1.434 (3)
P2—O21	1.5062 (16)	O13—C13	1.460 (3)
P2—N1	1.628 (2)	N1—C6	1.470 (3)
P2—C1	1.869 (2)	N1—C2	1.472 (3)
Cd1—O11	2.2256 (17)	C2—C3	1.517 (3)
Cd1—O11^i^	2.2256 (17)	C2—H2E	0.9900
Cd1—O21^i^	2.3173 (16)	C2—H2F	0.9900
Cd1—O21	2.3173 (16)	C3—H3E	0.9900
Cd1—O1	2.3409 (17)	C3—H3F	0.9900
Cd1—O1^i^	2.3409 (17)	C5—C6	1.517 (4)
Cd2—O12^ii^	2.1884 (17)	C5—H5E	0.9900
Cd2—O12	2.1884 (17)	C5—H5F	0.9900
Cd2—O3^ii^	2.2795 (16)	C6—H6E	0.9900
Cd2—O3	2.2795 (16)	C6—H6F	0.9900
Cd2—O2	2.3486 (16)	C13—H13A	0.9800
Cd2—O2^ii^	2.3486 (16)	C13—H13B	0.9800
O1—H1A	0.8439	C13—H13C	0.9800
			
O12—P1—O11	120.19 (11)	Cd2—O3—H3A	109.4
O12—P1—O13	109.73 (10)	Cd2—O3—H3B	118.2
O11—P1—O13	106.42 (10)	H3A—O3—H3B	107.4
O12—P1—C1	107.98 (10)	C3—O4—C5	110.07 (19)
O11—P1—C1	107.43 (10)	P1—O11—Cd1	132.97 (10)
O13—P1—C1	103.90 (10)	P1—O12—Cd2	165.00 (12)
O22—P2—O21	118.72 (10)	C13—O13—P1	121.93 (16)
O22—P2—N1	108.40 (10)	P2—O21—Cd1	133.58 (9)
O21—P2—N1	109.04 (10)	C6—N1—C2	111.82 (19)
O22—P2—C1	105.77 (10)	C6—N1—P2	124.48 (17)
O21—P2—C1	105.72 (10)	C2—N1—P2	123.28 (16)
N1—P2—C1	108.80 (11)	Cl1—C1—Cl2	108.31 (12)
O11—Cd1—O11^i^	166.14 (9)	Cl1—C1—P1	108.85 (12)
O11—Cd1—O21^i^	100.40 (6)	Cl2—C1—P1	108.55 (12)
O11^i^—Cd1—O21^i^	89.75 (6)	Cl1—C1—P2	107.53 (11)
O11—Cd1—O21	89.75 (6)	Cl2—C1—P2	107.98 (12)
O11^i^—Cd1—O21	100.40 (6)	P1—C1—P2	115.42 (12)
O21^i^—Cd1—O21	86.31 (8)	N1—C2—C3	109.5 (2)
O11—Cd1—O1	85.24 (6)	N1—C2—H2E	109.8
O11^i^—Cd1—O1	84.49 (6)	C3—C2—H2E	109.8
O21^i^—Cd1—O1	174.23 (5)	N1—C2—H2F	109.8
O21—Cd1—O1	95.00 (6)	C3—C2—H2F	109.8
O11—Cd1—O1^i^	84.49 (6)	H2E—C2—H2F	108.2
O11^i^—Cd1—O1^i^	85.24 (6)	O4—C3—C2	111.1 (2)
O21^i^—Cd1—O1^i^	95.00 (6)	O4—C3—H3E	109.4
O21—Cd1—O1^i^	174.23 (5)	C2—C3—H3E	109.4
O1—Cd1—O1^i^	84.25 (8)	O4—C3—H3F	109.4
O12^ii^—Cd2—O12	180.00 (9)	C2—C3—H3F	109.4
O12^ii^—Cd2—O3^ii^	82.50 (6)	H3E—C3—H3F	108.0
O12—Cd2—O3^ii^	97.50 (6)	O4—C5—C6	111.2 (2)
O12^ii^—Cd2—O3	97.50 (6)	O4—C5—H5E	109.4
O12—Cd2—O3	82.50 (6)	C6—C5—H5E	109.4
O3^ii^—Cd2—O3	180.00 (8)	O4—C5—H5F	109.4
O12^ii^—Cd2—O2	94.94 (6)	C6—C5—H5F	109.4
O12—Cd2—O2	85.06 (6)	H5E—C5—H5F	108.0
O3^ii^—Cd2—O2	92.88 (6)	N1—C6—C5	108.6 (2)
O3—Cd2—O2	87.12 (6)	N1—C6—H6E	110.0
O12^ii^—Cd2—O2^ii^	85.06 (6)	C5—C6—H6E	110.0
O12—Cd2—O2^ii^	94.94 (6)	N1—C6—H6F	110.0
O3^ii^—Cd2—O2^ii^	87.12 (6)	C5—C6—H6F	110.0
O3—Cd2—O2^ii^	92.88 (6)	H6E—C6—H6F	108.3
O2—Cd2—O2^ii^	180.0	O13—C13—H13A	109.5
Cd1—O1—H1A	117.7	O13—C13—H13B	109.5
Cd1—O1—H1B	118.1	H13A—C13—H13B	109.5
H1A—O1—H1B	101.1	O13—C13—H13C	109.5
Cd2—O2—H2A	112.6	H13A—C13—H13C	109.5
Cd2—O2—H2B	108.2	H13B—C13—H13C	109.5
H2A—O2—H2B	101.1		
			
O12—P1—O11—Cd1	82.84 (17)	O21—P2—N1—C2	163.49 (18)
O13—P1—O11—Cd1	−151.80 (14)	C1—P2—N1—C2	−81.7 (2)
C1—P1—O11—Cd1	−41.01 (18)	O12—P1—C1—Cl1	174.59 (11)
O11^i^—Cd1—O11—P1	151.44 (15)	O11—P1—C1—Cl1	−54.41 (14)
O21^i^—Cd1—O11—P1	−72.10 (16)	O13—P1—C1—Cl1	58.11 (13)
O21—Cd1—O11—P1	14.09 (16)	O12—P1—C1—Cl2	56.89 (14)
O1—Cd1—O11—P1	109.13 (16)	O11—P1—C1—Cl2	−172.11 (11)
O1^i^—Cd1—O11—P1	−166.20 (16)	O13—P1—C1—Cl2	−59.59 (13)
O11—P1—O12—Cd2	−39.7 (5)	O12—P1—C1—P2	−64.44 (15)
O13—P1—O12—Cd2	−163.4 (4)	O11—P1—C1—P2	66.56 (15)
C1—P1—O12—Cd2	83.9 (5)	O13—P1—C1—P2	179.07 (11)
O3^ii^—Cd2—O12—P1	77.6 (4)	O22—P2—C1—Cl1	−173.74 (11)
O3—Cd2—O12—P1	−102.4 (4)	O21—P2—C1—Cl1	59.52 (13)
O2—Cd2—O12—P1	−14.7 (4)	N1—P2—C1—Cl1	−57.47 (14)
O2^ii^—Cd2—O12—P1	165.3 (4)	O22—P2—C1—Cl2	−57.06 (14)
O12—P1—O13—C13	−18.2 (2)	O21—P2—C1—Cl2	176.19 (10)
O11—P1—O13—C13	−149.7 (2)	N1—P2—C1—Cl2	59.21 (14)
C1—P1—O13—C13	97.1 (2)	O22—P2—C1—P1	64.58 (14)
O22—P2—O21—Cd1	−84.54 (15)	O21—P2—C1—P1	−62.16 (14)
N1—P2—O21—Cd1	150.72 (12)	N1—P2—C1—P1	−179.15 (11)
C1—P2—O21—Cd1	33.90 (15)	C6—N1—C2—C3	55.4 (3)
O11—Cd1—O21—P2	−10.33 (14)	P2—N1—C2—C3	−131.8 (2)
O11^i^—Cd1—O21—P2	179.17 (13)	C5—O4—C3—C2	59.5 (3)
O21^i^—Cd1—O21—P2	90.11 (13)	N1—C2—C3—O4	−56.6 (3)
O1—Cd1—O21—P2	−95.53 (13)	C3—O4—C5—C6	−60.6 (3)
O22—P2—N1—C6	−155.22 (19)	C2—N1—C6—C5	−55.9 (3)
O21—P2—N1—C6	−24.6 (2)	P2—N1—C6—C5	131.4 (2)
C1—P2—N1—C6	90.2 (2)	O4—C5—C6—N1	58.1 (3)
O22—P2—N1—C2	32.9 (2)		

Symmetry codes: (i) −*x*+1, *y*, −*z*+1/2; (ii) −*x*+1, −*y*+1, −*z*.

### Hydrogen-bond geometry (Å, °)

**Table:**

*D*—H···*A*	*D*—H	H···*A*	*D*···*A*	*D*—H···*A*
O1—H1A···O2^iii^	0.84	2.06	2.849 (2)	156
O1—H1B···O22^iv^	0.88	2.12	2.990 (2)	170
O2—H2A···O21^i^	0.86	2.04	2.844 (2)	155
O2—H2B···O22	0.86	1.84	2.662 (2)	159
O3—H3A···O22	0.83	2.03	2.773 (2)	149
O3—H3B···O13^v^	0.90	1.87	2.745 (2)	163

Symmetry codes: (iii) −*x*+1, *y*−1, −*z*+1/2; (iv) *x*, *y*−1, *z*; (i) −*x*+1, *y*, −*z*+1/2; (v) *x*, *y*+1, *z*.
